# MiR-942-5p targeting the IFI27 gene regulates HCT-8 cell apoptosis via a TRAIL-dependent pathway during the early phase of *Cryptosporidium parvum* infection

**DOI:** 10.1186/s13071-022-05415-3

**Published:** 2022-08-16

**Authors:** Fujie Xie, Yajun Zhang, Juanfeng Li, Lulu Sun, Longxian Zhang, Meng Qi, Sumei Zhang, Fuchun Jian, Xiaoying Li, Junqiang Li, Changsheng Ning, Rongjun Wang

**Affiliations:** 1 College of Veterinary Medicine, Henan Agricultural University, Zhengzhou, 450046 China; 2 College of Animal Science, Tarim University, Alar, 843300 Xinjiang China

**Keywords:** *Cryptosporidium parvum*, HCT-8 cell, miR-942-5p, Apoptosis, Parasite burden

## Abstract

**Background:**

MicroRNAs (miRNAs) are involved in the regulation of both the innate and adaptive immune response to *Cryptosporidium parvum* infection. We previously reported that *C. parvum* upregulated miR‑942‑5p expression in HCT‑8 cells via TLR2/TLR4‑NF‑κB signaling. In the present study, the role of miRNA-942-5p in the regulation of tumor necrosis factor-related apoptosis-inducing ligand (TRAIL)-mediated HCT-8 cell apoptosis induced by *C. parvum* was investigated.

**Methods:**

Quantitative real-time polymerase chain reaction, western blotting, flow cytometry, and immunofluorescence were used for analysis.

**Results:**

Forced expression of miRNA-942-5p resulted in decreased apoptosis and an increased *C. parvum* burden in HCT-8 cells. The opposite results were observed using the suppressed expression of miRNA-942-5p. The miRNA-942-5p led to the translational suppression of IFI27 gene through targeting the 3’-untranslated region of the IFI27 gene. Moreover, overexpression of the IFI27 gene produced a high apoptotic ratio and low *C. parvum* burden. In contrast, a low apoptotic ratio and a high *C. parvum* burden were observed following downregulation of the IFI27 gene. Both miR-942-5p and the IFI27 gene influenced TRAIL and caspase-8 expression induced by *C. parvum* in HCT-8 cells. Moreover, TRAIL promoted HCT-8 cell apoptosis in a concentration-dependent manner.

**Conclusions:**

These data suggested that *C. parvum* induced the downregulation of IFI27 via relief of miR-942-5p-mediated translational suppression. IFI27 downregulation was affected the burden of *C. parvum* by regulating HCT-8 cell apoptosis through TRAIL-dependent pathways. Future studies should determine the mechanisms by which *C. parvum* infection increases miR-942-5p expression and the role of miR-942-5p in hosts' anti-*C. parvum* immunity in vivo.

**Graphical Abstract:**

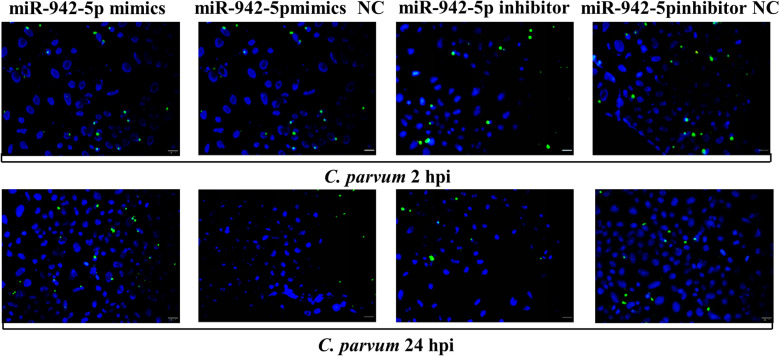

**Supplementary Information:**

The online version contains supplementary material available at 10.1186/s13071-022-05415-3.

## Background

Diarrheal disease is an important cause of malnourishment in children, which can enhance susceptibility to many other infections (e.g., malaria, pneumonia, and measles), resulting in higher mortality rates [1]. *Cryptosporidium* is an emerging zoonotic pathogen that causes diarrhea in both immunocompetent and immunosuppressed hosts and is second only to rotavirus as a cause of moderate-to-severe diarrhea in children < 2 years old [2]. There is extensive genetic variation within the genus *Cryptosporidium*. To date, at least 45 valid species and > 60 genotypes of *Cryptosporidium* have been identified or described [3], among which *C. parvum* and *C. hominis* are responsible for > 90% of infections in humans. Currently, although there are no fully efficacious treatment options or vaccines for cryptosporidiosis, nitazoxanide has been approved for the treatment of cryptosporidiosis in immunocompetent individuals [4]. However, the pathogenic mechanism of cryptosporidiosis remains largely unknown, which is a major factor limiting the availability of effective treatment.

Since *C. parvum* depends on the host cell for growth and development, apoptosis is considered an important defense mechanism against *Cryptosporidium* infection during innate immunity [5–8]. Two-phase modulation of apoptotic mechanisms was observed during cryptosporidiosis. The balance of apoptosis-associated gene expression was induced during the early phase of *Cryptosporidium* infection, with respect to antiapoptotic genes, whereas the balance was inhibited with respect to proapoptotic genes. The opposite effect was observed during the late stage of infection when proapoptotic genes were induced and antiapoptotic genes were inhibited [6]. Similar to the above results, a previous study demonstrated that *C. parvum* triggered host cell apoptosis in bystander uninfected biliary epithelial cells, which may limit the spread of infection. Meanwhile, nuclear factor kappa-light-chain-enhancer of activated B cells/nuclear-factor-of-kappa-light-polypeptide-gene-enhancer in B cell inhibitor (NF-κB/IκB) signaling was activated in infected biliary epithelia, which protected infected cells from death and facilitated parasite survival and propagation [7]. Although a panel of proteins connected with apoptosis has been identified using a micro-array in HCT-8 cells induced by *C. parvum* infection [8], only a few proteins (e.g., osteoprotegerin and survivin) have been elucidated in the role of regulation of host-cell apoptosis [9, 10].

Micro(mi)RNAs function in RNA silencing and the post-transcriptional regulation of gene expression [11]. A large number of studies have demonstrated that miRNAs play a critical role in the regulation of both innate and adaptive immunity in pathogen-host interactions [8, 12, 13]. Because *C. parvum* lacks the key components required for small RNA-mediated post-transcriptional gene silencing, it is an ideal model for investigating miRNA-mediated defenses against infection in epithelial cells [14]. In recent studies, at least seven host miRNAs have been thought to be associated with *Cryptosporidium* infection, including *let-7i*, miR-98, miR-513, miR-424, miR-503, miR-221, and miR-27b [15–22]. Thus, miRNAs may modulate the epithelial immune responses at every step of the innate immune response following *Cryptosporidium* infection, including the production of antimicrobial molecules, expression of cytokines/chemokines, release of epithelial cell-derived exosomes, and feedback regulation of immune homoeostasis [23].

Our previous study of miRNA expression in HCT-8 cells infected with *C. parvum* found that miR-942-5p, miR-181d, miR-3976, miR-18b-3p, miR-34b-5p, and miR-3591-3p may regulate apoptosis during the early phase of infection [24]. In a more recent study, we showed that *C. parvum* upregulated miR-942-5p expression in HCT-8 cells via the Toll-like receptor (TLR) 2/TLR4-NF-κB signaling pathway [25]. The present study investigated the mechanism of miR-942-5p in regulating HCT-8 cell apoptosis triggered by *C. parvum* infection, namely that the upregulation of miR-942-5p decreased HCT-8 cell apoptosis by targeting the IFI27 gene via the tumor necrosis factor-related apoptosis-inducing ligand (TRAIL) pathway and protected *C. parvum* from being cleared during the early stage of infection. It is worth noting that although the level of accuracy reflected by in vitro tests is debatable, it can provide critical reference for in vivo studies.

## Methods

### Cell culture and *C. parvum*

HCT-8 human ileocecal adenocarcinoma cells (American Type Culture Collection, Manassas, VA) were maintained in Dulbecco’s Modified Eagle’s Medium (DMEM) supplemented with 10% fetal bovine serum, 4 mmol/l l-glutamine, 100 U/ml penicillin, and 100 U/ml streptomycin at 37 °C in a 5% CO_2_ atmosphere. *Cryptosporidium parvum* subtype IIdA19G1 oocysts were maintained in infected neonatal calves and stored in a 2.5% K2Cr2O7 solution at 4 °C after purification. As previously described, the oocysts were placed in 0.25% trypsin and 0.75% sodium taurocholate for 1 h with mixing every 5 min, followed by incubation at room temperature for 30 min [26, 27], three washes in phosphate-buffered saline (PBS), and then were resuspended.

### Plasmid preparation and miRNA mimic/inhibitor synthesis

Two algorithms (Targetscan 4.2 at http://www.Targetscan.org; miRBD at http://www.mirbd.org) [28, 29] were used for analyzing miR-942-5p. After analysis, we found that miR-942-5p has complementarity with the IFI27 3'-UTR, whereas IFI27 is expressed in intestinal epithelial cells and associated with cell apoptosis [30]. To validate the predicted miRNA:mRNA interaction, we utilized a pmirGLO Dual-Luciferase miRNA target expression vector (pmirGLO-REPORT Luciferase vector) (Promega, USA) and miRNA mimics. Complementary DNA oligonucleotides containing the putative miR-942 target binding site within the 3’-UTR of human IFI27 and those that did not contain the 3’-UTR of human IFI27 were synthesized with flanking *Sac*I and *Xba*I restriction enzyme digestion sites, annealed, and ligated into the *Sac*I-*Xba*I sites of the pmirGLO-REPORT Luciferase vector. The pmirGLO vector expresses two luciferases: (1) the firefly luciferase (an experimental reporter that can be subjected to the effect of miRNA regulation) and Renilla luciferase (an internal control). The experimental plasmid, pmirGLO-IFI27-WT, contained a synthesized miR-942-5p:SP1 target region corresponding to the miR-942-5p binding site on the IFI27 3’-UTR. The negative control plasmid, pmirGLO-IFI27-MU, contained a synthesized mutated IFI27 3’-UTR designed based on the miR-942-5p:SP1 target region using the microRNA predictor. The sequences are presented in Additional file 1: Table S1. RNA products (GenePharma, China) were: miR-942-5p mimics (mimic endogenous miR-942-5p) and non-specific negative control mimics NC; miR-942-5p inhibitor (inhibit miR-942-5p) in cells; and non-specific negative control inhibitor NC. The Negative control had a proprietary sequence with no homology to any known mammalian gene. The sequences are presented in Additional file 1: Table S2.

### IFI27 gene manipulation

Human IFI27 cDNA (GenBank no.: NM_001130080) was amplified by PCR and subcloned into a pcDNA3.1 eukaryotic expression vector (pcDNA3.1-IFI27-OE). The overexpression of the IFI27 gene in HCT-8 cells was performed by transfection with a recombinant vector with Lipofectamine 3000 reagent (Invitrogen, Carlsbad, CA, USA) according to the manufacturer’s protocol. siRNA targeting IFI27 mRNAs were designed by the RiboBio (Guangzhou, China). The siRNA oligonucleotides had no significant overlap with homologous gene sequences. Nonspecific siRNA containing the same nucleotides in an irregular sequence was used as a control (Additional file 1: Table S2).

### Flow cytometry for cell apoptosis

Cells were washed with PBS and lysed using pancreatic enzymes prior to centrifugation at 1000 rpm for 5 min at room temperature. The centrifuged supernatant was removed, and the cells were resuspended in PBS for centrifugation at 1000 rpm for 5 min at room temperature. Using an Annexin V: FITC Apoptosis Detection Kit I (BD, USA), the supernatant was removed once again, and the cells were resuspended in 490 μl chilled 1 × binding buffer (cell concentration of 10^5^–10^6^/ml), to which 5 μl annexin V‐FITC and 5 μl PI were added. Cells were vortexed and incubated on ice for 10 min. Cell apoptosis was determined using flow cytometry (FCM).

### Immunofluorescence assay (IFA)

Cells were grown on slides for 2 h and 24 h and were subsequently removed and washed twice with PBS followed by A600FLR-20X. A Sporo-Glo™ Reagent-Only Kit (Waterborne, Inc., Clinical and Environment Parasitology, USA) was performed in accordance with manufacturer’s instructions. Finally, monolayers were washed three times with PBS, partially air-dried, and then cover slipped with mounting media containing DAPI (VECTASHIELD antifade mounting medium with DAPI; Vector Labs, USA). Monolayers were imaged using a differential-interference microscope (DIC) (Olympus, Japan). Quantification of the *C. parvum* burden was determined by counting the total number of FITC-labeled parasites within 200 × fields per infected well. Images of 50 microscope fields were quantified using Image J (https://imagej.nih.gov/ij/) [31].

### RT-qPCR analysis

RT-qPCR analyses were performed according to previously described research [32]. HCT-8 cells were washed three times with PBS before adding 1 ml TRIzol reagent (Invitrogen, USA) to each well. The total RNA was isolated following the manufacturer’s instructions after treatment with Recombinant DNase I (Takara, Japan). Next, 1 μg of extracted RNA was reverse transcribed with a ReverTra Ace qPCR RT Master Mix with gDNA Remover (Toyobo, Japan) in accordance with the manufacturer’s protocol. The levels of IFI27, Apaf-1, Caspase-8, TRAIL, and FasL messenger RNA, as well as *C. parvum* SSU rRNA, relative to those of the control genes human β-actin or SSU rRNA in HCT-8 cells were determined using real-time PCR with SYBR® Green Realtime PCR Master Mix (Toyobo, Japan). RT-PCR tests were performed using an ABI Quantstudio 7 detection system (Applied Biosystems, Thermo Fisher Scientific, USA). To compare the differences in the fold change of expression between and within experimental conditions, a representative sample was selected from the comparator condition (e.g. uninfected, initial time point group or vehicle-treated) and was used as a reference to calculate the ΔΔCq for each sample. Fold-change differences in gene expression were calculated using the 2^−ΔΔCq^ method. The primers are presented in Additional file 1: Table S1. This approach was used because it enables an objective look at the variability between samples in the control/reference group and it works well for studies in which the observations between groups are unpaired.

### Western blotting

Western blotting was utilized to analyze the level of IFI27 and TRAIL expression. The cells were lysed with a total protein extraction kit (Solarbio Life Sciences, China), and the protein concentrations were determined with a Pierce Bicinchoninic Acid (BCA) Assay Kit (Thermo Fisher Scientific, USA) in accordance with the manufacturer’s instructions. The lysed 30 μg protein samples were separated by 10% sodium dodecyl sulfate polyacrylamide gel electrophoresis (SDS-PAGE) and transferred onto polyvinylidene fluoride (PVDF) membranes. Following blocking with 1% bovine serum albumin (BSA), the membranes were incubated overnight at 4 °C with primary antibodies. The primary antibodies included rabbit anti-IFI27 (Abcam, UK), rabbit anti-TRAIL (Immunoway, USA), and rabbit anti-β-actin antibodies (Abcam, UK). The membranes were incubated with a horseradish peroxidase (HRP)-conjugated secondary antibody goat anti-rabbit antibody (Immunoway, USA) at room temperature for 2 h. The enhanced chemiluminescence (ECL) system was applied to visualize the protein bands, and the gray values of the protein bands were quantified using Image J software.

### Dual luciferase assay

Transfected cells were assayed using a Dual-GLO® Luciferase Assay System (Promega, USA). For each sample, the firefly and Renilla luciferase activities were measured sequentially by collecting emitted luminescence from the entire visible spectrum (300–700 nm) using a Synergy H1 Multi-Mode Reader (BioTek, USA). Briefly, the firefly luciferase activity was measured 10 min after induction of cell lysis and addition of the firefly luciferase substrate. We next quenched the firefly luciferase reaction and addition of the Renilla luciferase substrate. After 10 min, the Renilla luciferase activity was captured.

### Statistical analysis

Statistical analysis was performed using GraphPad Prism 8.0.2 (GraphPad Software, Inc., USA). *P*-values were calculated using an unpaired *t*-test with a Bonferroni correction between two groups or a nonparametric one-way ANOVA among multiple groups. All data are expressed as the mean ± SD. All experiments were performed on no less than three biological replicates. For all analyses, a threshold of *P* < 0.05 was considered significant.

## Results

### The miRNA-942-5p is associated with HCT-8 cell apoptosis and the *C. parvum* burden

**Fig. 1:**
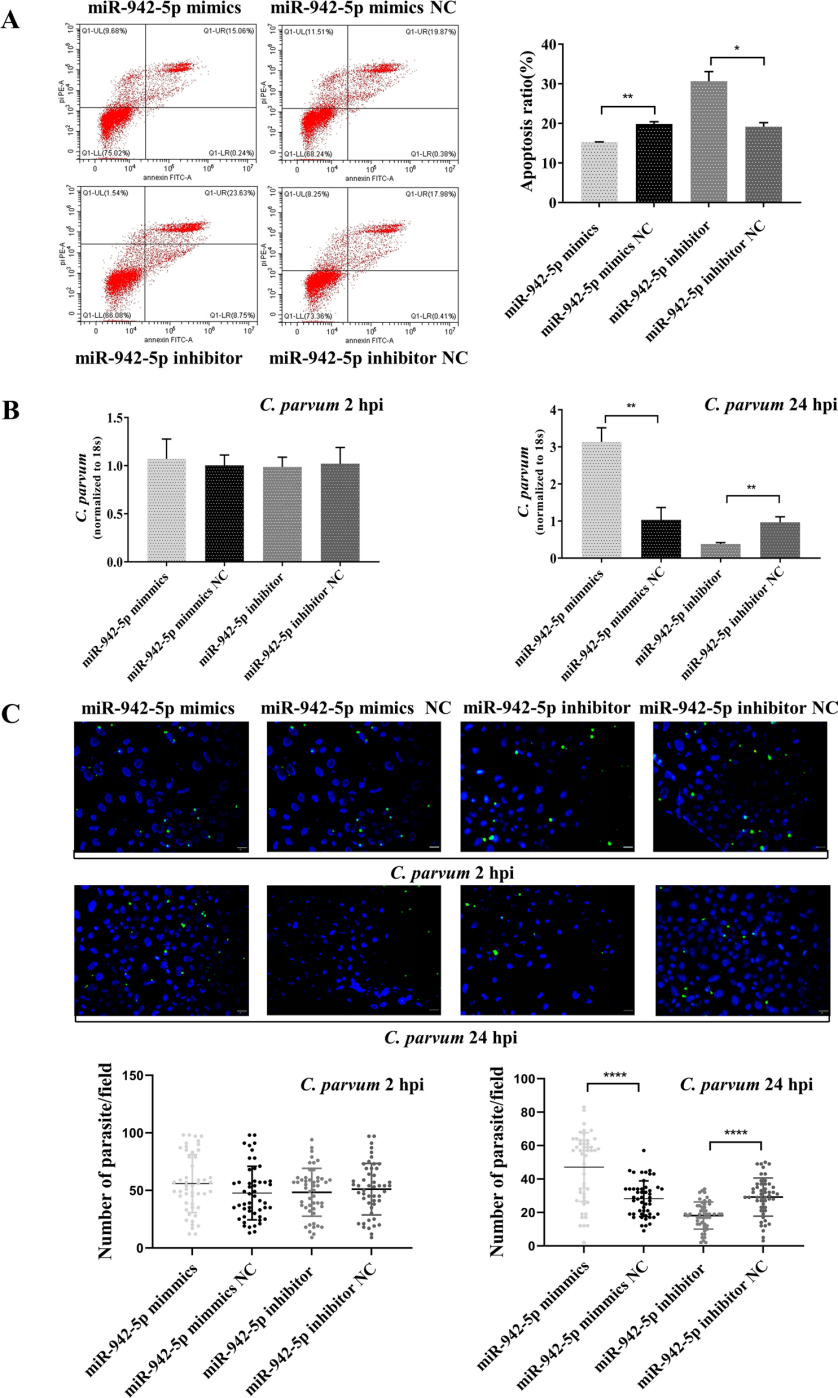
miR-942-5p is involved in cell apoptosis and parasite eradication during the early period following *C. parvum* infection. **A** The effect of miR-942-5p on cell apoptosis in HCT-8 cells. Cellular apoptosis was suppressed after transfection with miR-942-5p mimics, whereas apoptosis was enhanced after transfection with an miR-942-5p inhibitor. **B** and **C** The effect of miR-942-5p on the number of parasites in HCT-8 cells. The burden of *C. parvum* infection was significantly increased after transfection with an miR-942-5p mimic, whereas it was suppressed after in vitro transfection with an miR-942-5p inhibitor 24 h after initial parasite exposure. *Cryptosporidium parvum* parasites were stained in green, and the cell nuclei were stained blue. Bar = 20 µm. All data represent the combined mean ± SD of three independent experiments, with two to three technical replicates per experiment, and were analyzed with a *t*-test compared to cells transfected with a non-specific control. ^*^P < 0.05; ^**^P ≤ 0.01; ^***^P ≤ 0.001; ^****^P ≤ 0.0001

The cells were transfected with a specific miR-942-5p mimic and miR-942-5p inhibitor (100 nM, synthetic company recommended concentration) and 6 h later exposed to a constant number of *C. parvum* sporozoites for 2 h and then washed twice with PBS as a model for testing parasite attachment and cellular invasion. Following infection for 24 h, parasite proliferation in cells and the cellular apoptosis rate were tested as a reflection of the host antimicrobial defense. We detected a significantly lower apoptosis ratio following transfection with the miR-942-5p mimic, whereas the cells transfected with the miR-942-5p inhibitor had the opposite result (Fig. 1A). No changes in the parasite burden were detected in two groups for 2 hpi, which suggested that miR-942-5p did not affect the initial parasite host cell attachment and cellular invasion. We detected a significantly higher parasite burden in the cells transfected with the miR-942-5p mimic and a low parasite burden in the miR-942-5p inhibitor group at 24 hpi, which was confirmed by qPCR and a immunofluorescence assay (Fig. 1B and 1C).

### *Cryptosporidium parvum* infection increased miR-942-5p expression, resulting in IFI27 translational suppression

**Fig. 2:**
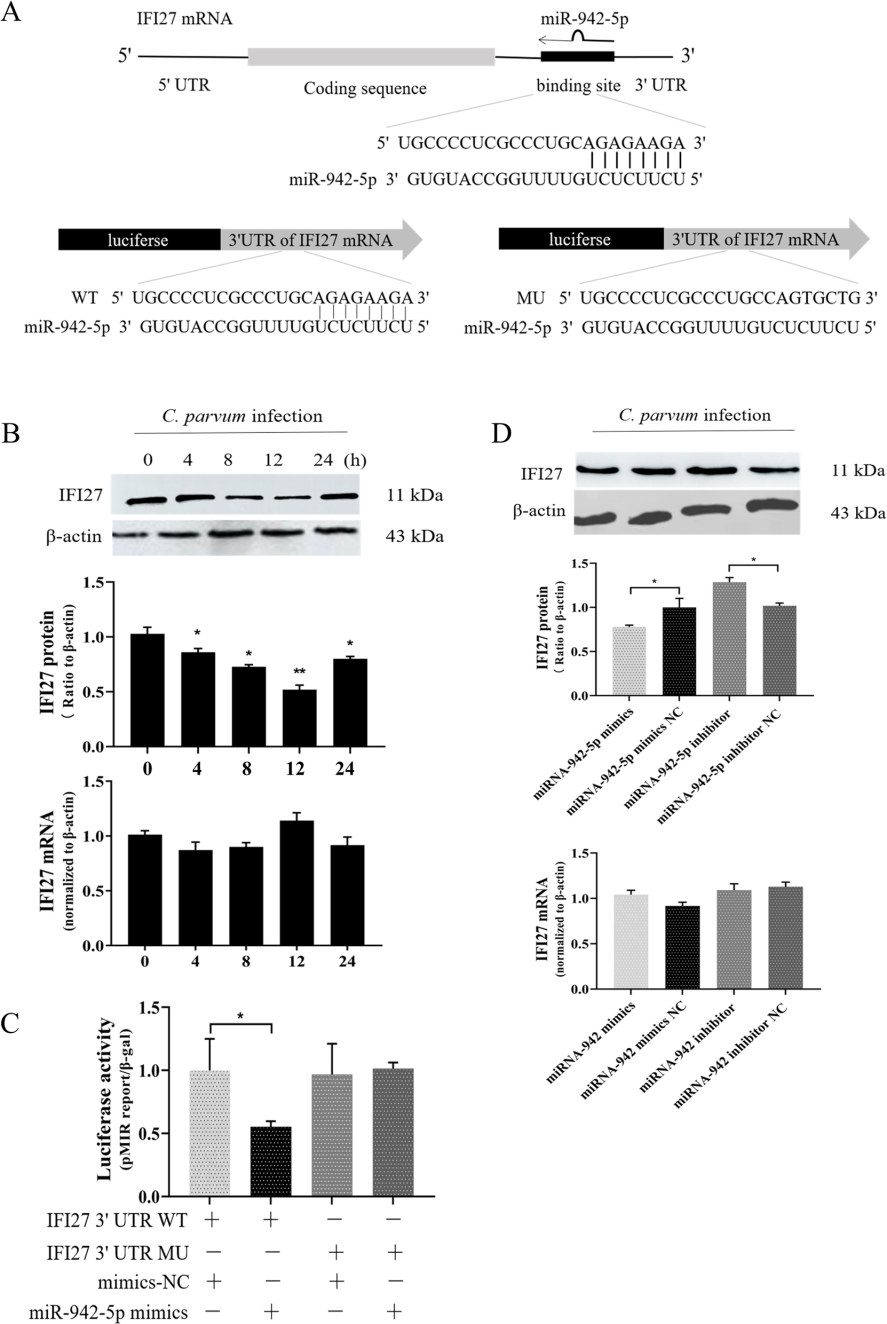
The miR-942-5p targets the IFI27 3′-UTR, causing translational suppression. **A** The IFI27 mRNA schematic showed one potential binding site for miR-942-5p in its 3′-UTR. The IFI27 3′-UTR sequence covering the potential binding site was inserted into the pmirGLO-REPORT luciferase plasmid. Control plasmids with the mutant 3′-UTR sequence were also generated as a control. **B** HCT-8 cells were exposed to *C. parvum* sporozoites for as long as 24 h, followed by western blot to evaluate IFI27 protein expression and real-time PCR analysis for IFI27 mRNA expression. Representative western blots from three independent experiments are shown. The densitometric levels of protein signals and mRNA levels were quantified and expressed as the ratio to actin. **C** Targeting the IFI27 3′-UTR resulted in translational suppression. Cells were transfected with a pMIR-REPORT luciferase construct containing the miR-942-5p binding site in the IFI27 3′-UTR and treated with an miR-942-5p mimic for 24 h, followed by a luciferase analysis. **D** Manipulation of miR-942-5p function can result in reciprocal alterations in IFI27 protein expression in HCT-8 cells. Cells were treated with an miR-942-5p mimic or miR-942-5p inhibitor for 6 h and exposed to *C. parvum* sporozoites, followed by a western blot for IFI27 protein and real-time PCR for IFI27 mRNA 12 hpi. Representative western blot images and quantification of IFI27 mRNA levels from three independent experiments are shown. Densitometric levels of IFI27 signals were quantified and expressed as the ratio to β-actin. Densitometric levels of protein signals and mRNA levels were quantified and expressed as the ratio to β-actin. All data represent the combined mean ± SD of three independent experiments, with two to three technical replicates per experiment, and were analyzed with a *t*-test vs. the controls. ^*^P < 0.05; ^**^P ≤ 0.01

miR-942-5p displays complementarity with IFI27 3’-UTR (Fig. 2A). To elucidate whether miR-942-5p can bind to the IFI27 3’-UTR and result in translational suppression, we generated pmiGLO-REPORT luciferase constructs containing the IFI27 3’-UTR with the putative miR-942-5p binding site (Fig. 2A). The pmiGLO-REPORT luciferase constructs containing the IFI27 3'-UTR with a mutation at the putative miR-942-5p binding site (AGAGAAGA to CAGTGCTG) were generated as controls (Fig. 2A). We measured the levels of IFI27 protein expression in HCT-8 cells following exposure to *C. parvum* sporozoites for up to 24 h. Since we wanted to see how the protein level of IFI27 changed after cells were infected with *C. parvum*, we took 0 h as the infection control. A significant decrease in the levels of IFI27 protein expression was detected in the cells infected with *C. parvum* for 8 h and 12 h (Fig. 2B). No significant changes in the levels of IFI27 mRNA expression were detected by real-time PCR (Fig. 2B). The cells were co-transfected with firefly luciferase constructs containing the wild type or mutant 3'-UTR of the putative miR-942-5p target, miR-942-5p mimic, or NC using Lipofectamine 3000, followed by an assessment of luciferase activity at 48 h after transfection. As shown in Fig. 2C, a significant decrease in luciferase activity was detected in the cells transfected with the miR-942-5p mimic after transfection with the IFI27 3'-UTR construct containing the putative binding site compared with NC cells transfected with the miR-942-5p mimic. No changes in luciferase activity were observed in the cells transfected with the mutant IFI27 3'-UTR construct.

To test whether miRNA-mediated translational repression of IFI27 was relevant to IFI27 protein expression, we treated HCT-8 cells with an miR-942-5p mimic or miR-942-5p inhibitor for 6 h after the addition of *C. parvum* sporozoites. The level of IFI27 mRNA and protein expression was evaluated by qPCR and western blots. The transfection of HCT-8 cells with the miR-942-5p mimic caused a decrease in IFI27 protein content. No significant changes in the level of IFI27 mRNA expression was found between the control cells and those treated with the miR-942-5p mimic. In contrast, an increase in the IFI27 protein content was identified in the cells treated with the miR-942 inhibitor. No significant change in the level of IFI27 mRNA expression was found between the control cells and those treated with an miR-942-5p inhibitor (Fig. 2D). Since our previous study found that miR-942-5p was strongly upregulated during the early phase of *C. parvum* infection by the microarray analysis [24], these data suggest that *C. parvum* infection increased miR-942-5p expression resulting in suppressed endogenous translation of IFI27 by acting on the domain of IFI27 3'-UTR.

### IFI27 is involved in the regulation of HCT-8 cell apoptosis and *C. parvum* burdens

**Fig. 3:**
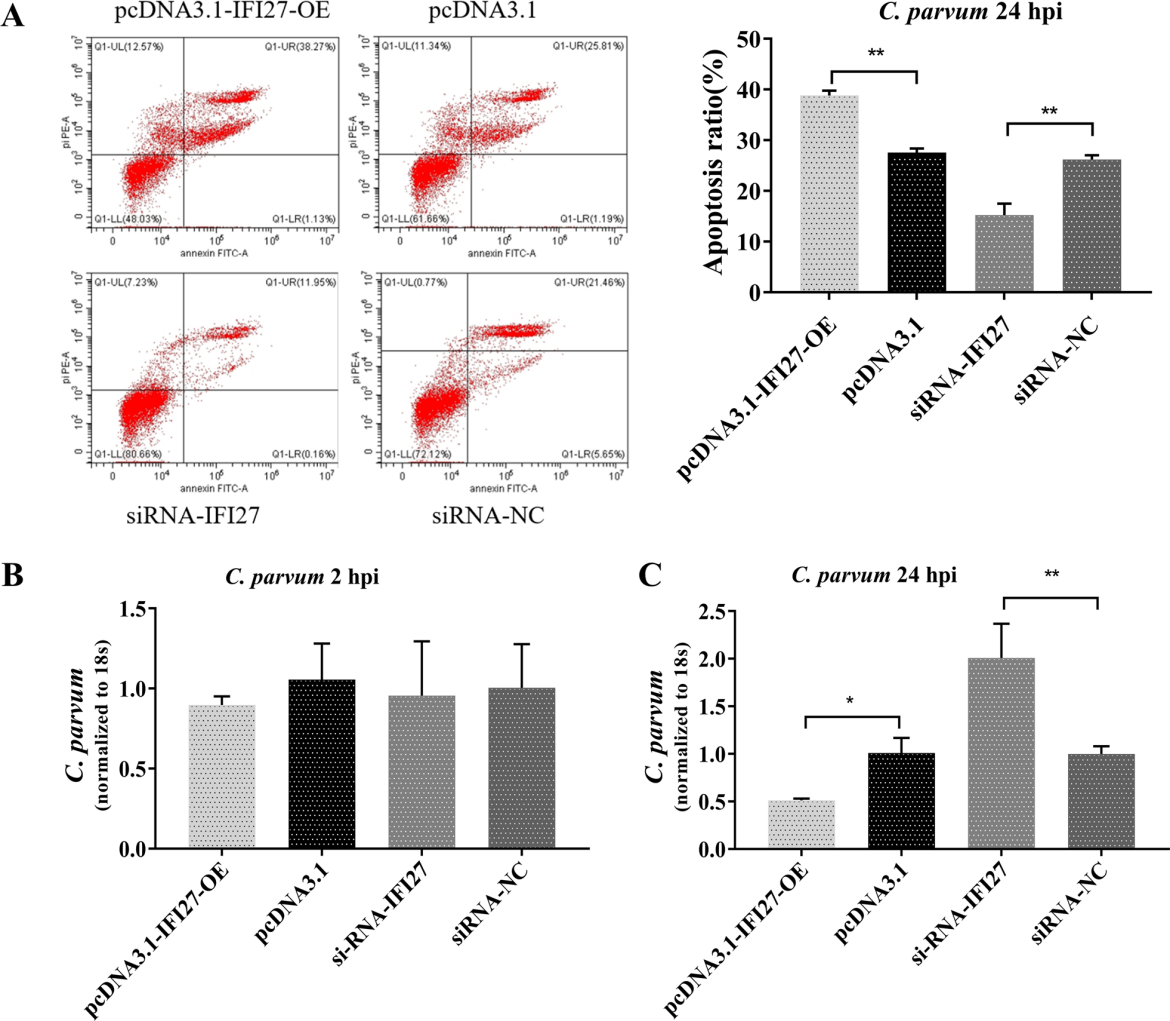
IFI27 is involved in cell apoptosis and parasite eradication following early *C. parvum* infection. **A** Effect of IFI27 on cell apoptosis in HCT-8 cells. The cells were transfected with pcDNA3.1-IFI27-OE or siRNA-IFI27 for 24 h. Cells were exposed to an equal number of *C. parvum* sporozoites for 24 h after digestion and staining, and the cell apoptosis ratio was assessed by flow cytometry. Cellular apoptosis was enhanced after transfection with pcDNA3.1-IFI27-OE, but was restrained after transfection with siRNA-IFI27. **B**, **C** Effect of IFI27 on the number of parasites in HCT-8 cells. Cells were transfected with pcDNA3.1-IFI27-OE or siRNA-IFI27 for 24 h. Cells were exposed to an equal number of *C. parvum* sporozoites for 2 h, followed by extensive washing with culture medium. To determine the initial attachment and cellular invasion of *C. parvum*, the cells were immediately harvested after washing and *C. parvum* was quantified by real-time PCR. A similar number of parasites was detected in cells transfected with pcDNA3.1-IFI27-OE or siRNA-IFI27 following the initial exposure to *C. parvum* for 2 h. To determine the parasite burden after the initial cell attachment and invasion, infected HCT-8 cells were cultured for another 22 h after washing, followed by real-time PCR analysis. The *C. parvum* infection burden was obviously suppressed after transfection with pcDNA3.1-IFI27-OE but was increased after transfection with siRNA-IFI27 in vitro 24 h after the initial parasite exposure. All data represent the combined mean ± SD of three independent experiments with two to three technical replicates per experiment. The data were analyzed with a *t*-test and compared to cells transfected with an empty vector or nonspecific control siRNA. ^*^P < 0.05; ^**^P ≤ 0.01

To study whether miR-942 affected cellular apoptosis and the parasite burden by the translational suppression of IFI27, we constructed an IFI27 overexpression vector and selected an appropriate transfection concentration (100 nM) to upregulate the level of IFI27 protein expression in HCT-8 cells (Additional file 2: Fig. S1). We also designed three siRNAs targeting IFI27 to downregulate the IFI27 protein and the most appropriate transfection concentration (100 nM), and effective siRNA were selected (Additional file 2: Fig. S1). At 24 h following the initial infection, a significantly higher parasite burden and a lower apoptosis ratio were detected in the cells transfected with siRNA-IFI27 compared with the negative control, whereas the cells transfected with the pcDNA3.1-IFI27-OE had the opposite result (Fig. 3A and 3C). No changes of parasite burden were detected in cells transfected with siRNA-IFI27 and in cells transfected with the pcDNA3.1-IFI27-OE compared with their control groups after the cells were exposed to a constant number of *C. parvum* sporozoites for 2 h. These results suggested that IFI27 did not affect initial parasite host cell attachment and cellular invasion (Fig. 3C). Thus, IFI27 appeared to be involved in the regulation of cell apoptosis and the parasite burden in HCT-8 cells.

### IFI27 induces cellular apoptosis through the TRAI/TRAIL-mediated pathway

**Fig. 4:**
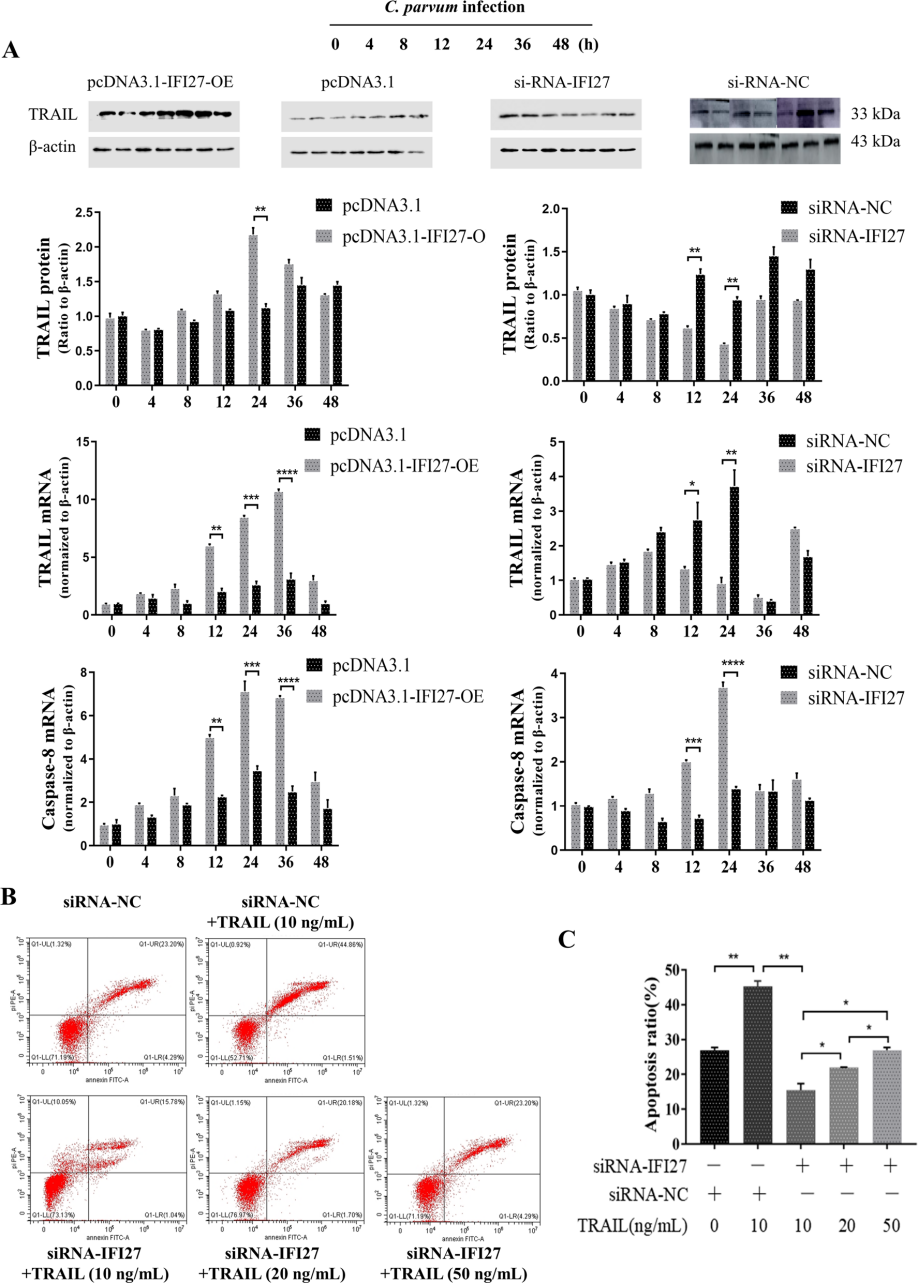
IFI27 is involved in cell apoptosis and parasite eradication following early *C. parvum* infection via a TRAIL-mediated pathway. **A** The effect of IFI27 on apoptosis-related molecules in HCT-8 cells. The cells were transfected with pcDNA3.1-IFI27-OE or siRNA-IFI27 for 24 h. Cells were exposed to an equal number of *C. parvum* sporozoites for up to 48 h, followed by a western blot for TRAIL protein expression and real-time PCR analysis for TRAIL and caspase-8 mRNA. Apoptosis-related molecules were enhanced after transfection with pcDNA3.1-IFI27-OE, but were restrained after transfection with siRNA-IFI27. **B**, **C** The effect of rTRAIL on cell apoptosis in HCT-8 cells. Cells were transfected with siRNA-IFI27 for 24 h. The cells were exposed to an equal number of *C. parvum* sporozoites for 2 h, followed by an incubation with varying concentrations of recombinant TRAIL (10, 25, and 50 ng/ml). After 6 h, the cells were digested and stained, and the apoptosis ratio was assessed by flow cytometry. With the increased concentration, the proportion of apoptosis was gradually increased. All data represent the combined mean ± SD of three independent experiments with two to three technical replicates per experiment. The data were analyzed with a one-way ANOVA followed by a Dunnett’s test for multiple comparisons. ^*^P < 0.05; ^**^P ≤ 0.01; ^***^P ≤ 0.001; ^****^P ≤ 0.0001

During the early stages after *C. parvum* infection, the expression of molecules related to cell apoptosis were detected. We found that the expression of several molecules was upregulated following infection with *C. parvum* in HCT-8 cells, including FasL, TRAIL, Apaf-1, and Caspase-8 (Additional file 3: Fig. S2). To further explore which pathway IFI27 regulated cell apoptosis, we transfected pcDNA3.1-IFI27-OE and siRNA-IFI27 into cells and exposed them to *C. parvum* sporozoites. Cellular apoptosis-related molecules were detected from 0 to 48 h by qPCR and western blots. We found that IFI27 overexpression led to an elevation in the level of TRAIL and caspase-8 expression compared with the control group (Fig. 4A). Downregulation in the level of TRAIL and caspase-8 expression was found after reducing the level of IFI27 expression compared with the control group (Fig. 4A). No changes were identified in the level of Apaf-1and FasL compared with the negative control (Additional file 4: Fig. S3).

Under pathological conditions, TRAIL becomes a potent inducer of apoptosis in intestinal epithelial cells. We speculated that IFI27 may regulate cell apoptosis throughout a TRAIL-mediated pathway following exposure to *C. parvum*. To test this hypothesis, we transfected siRNA-IFI27 into HCT-8 cells and infected the cells with *C. parvum*. The infected cells were treated with various concentrations of recombinant TRAIL (10, 25, and 50 ng/ml). After 6 h, the cell apoptosis ratio was assessed in all cultures. A low concentration could increase the cell apoptosis ratio following transfection with the siRNA-IFI27 negative control, and increased cell apoptosis ratio indicated that TRAIL enhanced cellular apoptosis. Furthermore, the addition of TRAIL increased the level of apoptosis in a dose-dependent manner after siRNA-IFI27 transfection (Fig. 4B and 4C).

### MiR-942 regulates TRAIL and caspase-8 expression after *C. parvum* infection in HCT-8 cells

**Fig. 5:**
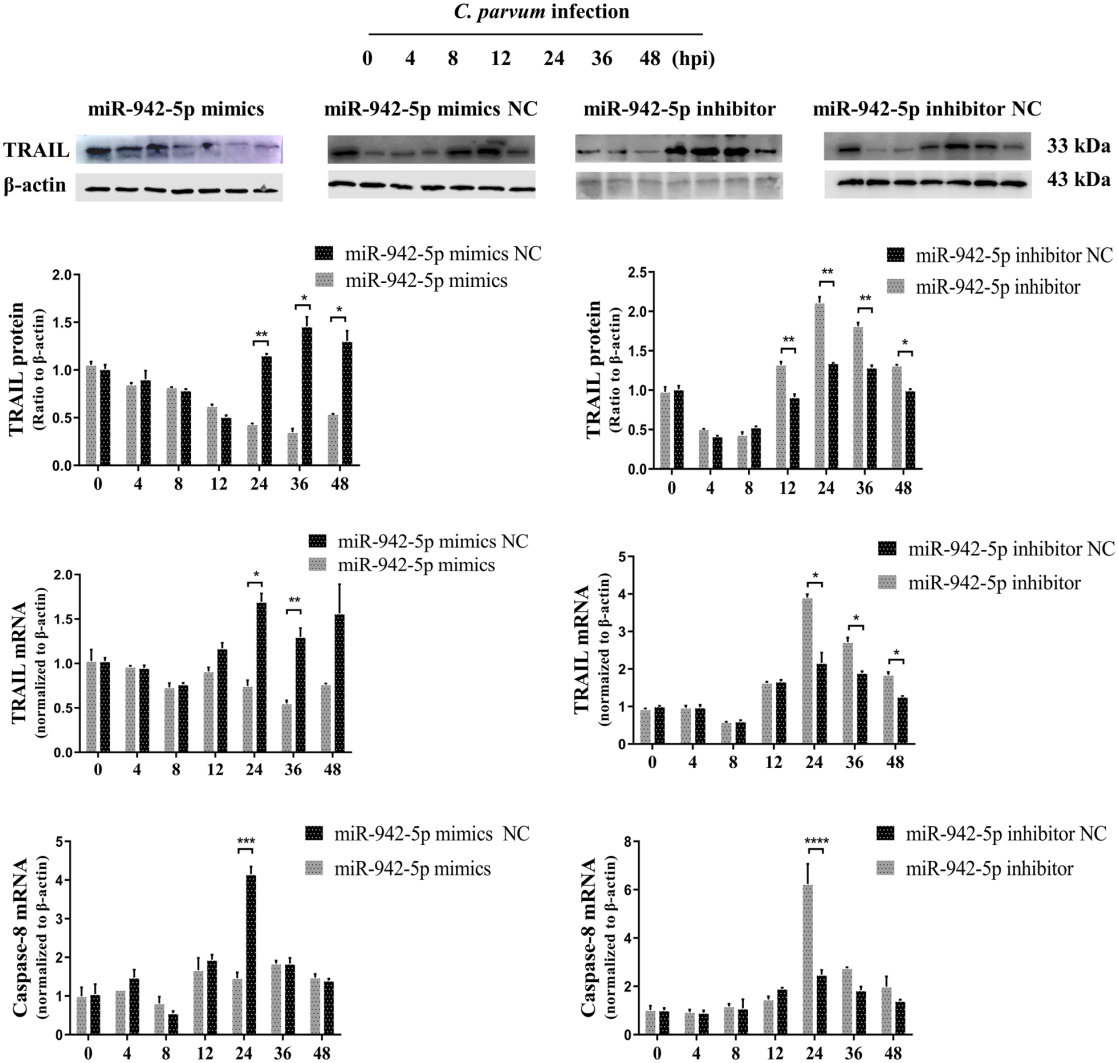
The miR-942-5p regulates TRAIL expression after *C. parvum* infection in HCT-8 cells. Cells were transfected with an miR-942-5p mimic or miR-942-5p inhibitor for 24 h. Cells were exposed to an equal number of *C. parvum* sporozoites for up to 48 h, followed by a western blot for TRAIL protein and real-time PCR analysis for TRAIL and caspase-8 mRNA. All data represent the combined mean ± SD of three independent experiments with two to three technical replicates per experiment. The data were analyzed with a one-way ANOVA followed by a Dunnett’s test for multiple comparisons. ^*^P < 0.05; ^**^P ≤ 0.01; ^***^P ≤ 0.001; ^****^P ≤ 0.0001

After transfection with an miR-942-5p mimic or miR-942-5p inhibitor, the cells were exposed to *C. parvum* sporozoites, after which TRAIL and caspase-8 expression was assessed by western blots and/or qPCR up to 48 h. We found that both TRAIL and caspase-8 expression were significantly decreased at 12 h and/or 24 h following transfection the miR-942-5p mimic compared to the control group. As expected, transfection with the miR-942-5p inhibitor was associated with a crosscurrent on the expression of TRAIL and caspase-8 compared to the control group at 24 h and/or 36 h, as expected (Fig. 5).

## Discussion

The miRNAs have been implicated in the resistance to *Cryptosporidium* infection. In biliary epithelial cells, a greater epithelial defense response against *C. parvum* was observed through the upregulation of TLR4 expression regulated by *let-7i* [15]. Moreover, *let-7i* functions together with miR-98 to control the expression of suppressors of inflammatory cytokine signaling (SOCS/CIS) and proteins [15, 17]. The miR-27b directly targets KSRP4 and modulates iNOS (inducible) mRNA stability following *C. parvum* infection [22]. Histone deacetylases (HDACs) have been described as regulators of miR-424 and miR-503 suppression, which in turn promote the mucosal anti-*C. parvum* defense [21]. Intercellular adhesion molecule-1 (ICAM-1) was described as the direct target of miR-221, and the downregulation of miR-221 induced by *C. parvum* is likely involved in the increased infiltration of lymphocytes into the intestinal mucosa [19]. B7-H1 expression is regulated by miR-513 and is involved in cholangiocyte-T cell interactions during *C. parvum* infection [20]. In this study, the upregulation of miR-942-5p induced by *C. parvum* resulted in the downregulation of IFI27 gene, which contributed to parasite survival during the early phase of infection by regulating HCT-8 cell apoptosis, representing a novel epithelial defense response mechanism against *C. parvum* infection.

*Cryptosporidium* spp. can improve their ability to infect and proliferate inside its target cells (i.e., epithelial cells) via inhibition of the host immune responses, including those involved in apoptosis and cytokine production [33, 34]. A microarray analysis of 51 apoptosis-associated genes demonstrated an anti-apoptotic state at 6 and 12 hpi and a moderately proapoptotic state at 24, 48, and 72 hpi [34]; however, little is known about the regulatory role of these apoptosis-associated genes at different stages of infection. A previous study suggested that *C. parvum* can actively inhibit apoptosis by upregulating survivin to favor its infection in intestinal epithelial cells [10]. In the present study, the IFI27 gene targeted by miR-942-5p was confirmed as a regulator of HCT-8 cell apoptosis in a TRAIL-dependent manner, indicating a new epithelial defense response mechanism against *C. parvum* infection. Previously, two extrinsic apoptosis pathways, Fas/FasL and TRAI/TRAIL, have been reported to be involved in the regulation of host-cell apoptosis induced by *Cryptosporidium* infection [9, 34–36].

The miR-942-5p may elicit a variety of biological regulation functions, including cell apoptosis. Indeed, previous studies suggest that miR-942-5p can regulate cellular apoptosis in response to microbial infection. For example, miR-942-5p downregulation enhances the apoptosis of HLCZ01 cells in response to hepatitis C virus infection [37]. Another study demonstrated that miR-942 decreased TRAIL-induced apoptosis through ISG12a (IFI27) downregulation and was regulated by AKT [38]. Similarly, IFI27 is an important protein that is involved in cell apoptosis, autophagy, oncolytic action, immune regulation, and other biological functions [39, 40]. The function of IFI27 is further illustrated in the case of viral infection. For example, after Newcastle disease virus (NDV) infection, host cells expressed high levels of IFI27, which promoted cell apoptosis by promoting the migration of Bax protein to mitochondria, thereby hindering NDV replication [41]. A recent microarray analysis of the intestinal epithelial cells transcriptional response in a study based on a *C. parvum* neonatal piglet infection model showed that the expression of the IFI27 gene at peak infection (days 3–5) was significantly upregulated [42], while another study showed that a significant elevation in IF127 gene expression in human small intestinal enteroids at 72 h of infection but interestingly not at 24 h [43]; however, the role of IFI27 gene upregulation has not been elucidated. We must admit that utilizing ileocecal adenocarcinoma cells has certain limitations. A previous study suggested that the activity of caspase-3 was detected in villous epithelium of piglet ileum after *C. parvum* infection; however, the expression of X-linked inhibitor of apoptosis protein (XIAP) was also upregulating, thus inhibiting apoptosis and ensuring organism integrity of barrier function [44].

In conclusion, our data indicated that *C. parvum* induced IFI27 downregulation in HCT-8 cells via relief of miR-942-5p-mediated translational suppression. Moreover, IFI27 downregulation was involved in the *C. parvum* burden by regulating cell apoptosis through a TRAIL-dependent pathway during the early phase of infection. Thus, miR-942-5p played a role in regulating IFI27 expression in response to *C. parvum* infection in HCT-8 cells. Future studies should determine the mechanism by which *C. parvum* infection increases miR-942-5p expression and the role of miRNAs in host anti-*C. parvum* immunity in vivo.

## Supplementary Information

**Additional file 1: Table S1**. Primers used in RT-qPCR and sequences used for construct generating.** Table S2**. RNA oligonucleotides for miRNA and siRNA.

**Additional file 2: Figure S1.** (A) Screening of siRNA transfection concentration, the transfectant was positive GAPDH siRNA. The optimal transfection concentration was 100 nM. (B) Screening of siRNA. si-IFI27-2 worked best. (C) Screening of plasmid transfection concentration. The optimal transfection concentration was 100 nM. All data represent the combined mean ± SD of three independent experiments with three technical replicates per experiment and were analyzed with a *t*-test vs. the controls. *P < 0.05; **P ≤ 0.01.

**Additional file 3: Figure S2.** The level of apoptosis-related molecule expression at different time points after *C. parvum* infection. Cells were exposed to an equal number of C. parvum sporozoites for up to 48 h, followed by real-time PCR analysis for FasL, TRAIL, Apaf-1, and caspase-8 mRNA. All data represent the combined mean ± SD of three independent experiments with two to three technical replicates per experiment. The data were analyzed with a one-way ANOVA followed by a Dunnett’s test for multiple comparisons. *P < 0.05; **P ≤ 0.01; ***P ≤ 0.001; ****P ≤ 0.0001.

**Additional file 4: Figure S3.** IFI27 does not regulate FasL and Apaf-1 expression after C. parvum infection in HCT-8 cells. Cells were transfected with pcDNA3.1-IFI27-OE or siRNA-IFI27 for 24 h. Cells were exposed to an equal number of *C. parvum* sporozoites for up to 48 h, followed by real-time PCR analysis for FasL and Apaf-1 mRNA. All data represent the combined mean ± SD of three independent experiments with two to three technical replicates per experiment. The data were analyzed with a one-way ANOVA followed by a Dunnett’s test for multiple comparisons.

## Data Availability

Data are available from the authors upon reasonable request.

## Abbreviations

*C. parvum*: *Cryptosporidium parvum*
NF-κB: Nuclear factor kappa-light-chain-enhancer of activated B cells
IκB: Nuclear factor of kappa light polypeptide gene enhancer in B cells inhibitor
TLR: Toll-like receptor
TRAIL: Numor necrosis factor-related apoptosis-inducing ligand
DMEM: Dulbecco’s Modified Eagle’s Medium
FCM: Flow cytometry
PBS: Phosphate-buffered saline
BCA: Bicinchoninic Acid
SDS-PAGE: Sulfate polyacrylamide gel electrophoresis
PVDF: Polyvinylidene fluoride
BSA: Bovine serum albumin
ECL: Enhanced chemiluminescence
