# Supplementary material for: MiR-942-5p targeting the IFI27 gene regulates HCT-8 cell apoptosis via a TRAIL-dependent pathway during the early phase of Cryptosporidium parvum infection
Source: Parasit Vectors. 2022 Aug 16;15:291. doi: 10.1186/s13071-022-05415-3 (PMC9382849; doi:10.1186/s13071-022-05415-3)
Supplement: Supplementary file 1 — Additional file 1: Table S1. Primers used in RT-qPCR and sequences used for construct generating. Table S2. RNA oligonucleotides for miRNA and siRNA. [file 13071_2022_5415_MOESM1_ESM.docx]

**Table S1.** Primers used in RT-qPCR and sequences used for construct generating

| **Target** | **Primers** |  |
| --- | --- | --- |
|  | **Forward** | **Reverse** |
| Human-SSU rRNA | TAG AGA TTG GAG GTT GTT CCT | CTC CAC CAA CTA AGA ACG GCC |
| *C. parvum-*SSU rRNA | CCG ATA ACG AAC GAG ACT CTG G | TAG GGT AGG CAC ACG CTG AGC C |
| IFI27 | TGC CAT GGG CTT CAC TGC GG | CTG CCC GAG GCA ACT CCA CC |
| TRAIL | TGC GTG CTG ATC GTG ATC TTC | GCT CGT TGG TAA AGT ACA CGT A |
| FasL | TCC GAG AGT CTA CCA GCC A | CAT TCC AGA GGC ATG GAC CTT |
| Apaf-1 | AAT GGC AGG CTG TGG GAA GTC | TAA GTG GAA GCC TCT GGG AAA AAC |
| Caspase-8 | TTT CTG CCT ACA GGG TCA TGC | CTG TAC CAG ACC GAG ATG TCA |
| β-actin | AGC GAG CAT CCC CCA AAG TT | GGG CAC GAA GGC TCA TCA TT |
| pcDNA3.1-IFI27-OE^a^ | CCC aagctt GCC ACC ATG GAG GCC TCT GCT CTC AC | CCG gaattc GTA GAA CCT CGC AAT GAC AGC |
| **DNA oligonucleotides for construct** | **Sequences** | |
| pmirGLO luciferase construct containing IFI27 3' UTR | CTG CCC CTC GCC CTG CAG AGA AGA TGC CCC TCG CCC TGC AGA GAA GAT GCC CCT CGC CCT GCT | |
| pmirGLO luciferase construct containing IFI27 3’ UTR with mutation | CTG CCC CTC GCC CTG CCA GTG CTG TGC CCC TCG CCC TGC CAG TGC TGT GCC CCT CGC CCT GCT | |

^a^ Restriction enzyme sites were indicated by lower case letters.

**Table S2.** RNA oligonucleotides for miRNA and siRNA

| **Target** | **Sequences** | |
| --- | --- | --- |
| miR-942-5p mimics | UCU UCU CUG UUU UGG CCA UGU G | CAU GGC CAA AAC AGA GAA GAU U |
| miR-942-5p mimics NC | UUC UCC GAA CGU GUC ACG UTT | ACG UGA CAC GUU CGG AGA ATT |
| miR-942-5p inhibitor | CAC AUG GCC AAA ACA GAG AAG A |  |
| miR-942-5p inhibitor NC | CAG UAC UUU UGU GUA GUA CAA |  |
| siRNA-IFI27 | AGT GAC UGC AGA GUA GCC A |  |
