# Supplementary figures and images for: MiR-942-5p targeting the IFI27 gene regulates HCT-8 cell apoptosis via a TRAIL-dependent pathway during the early phase of Cryptosporidium parvum infection
Source: Parasit Vectors. 2022 Aug 16;15:291. doi: 10.1186/s13071-022-05415-3 (PMC9382849; doi:10.1186/s13071-022-05415-3)

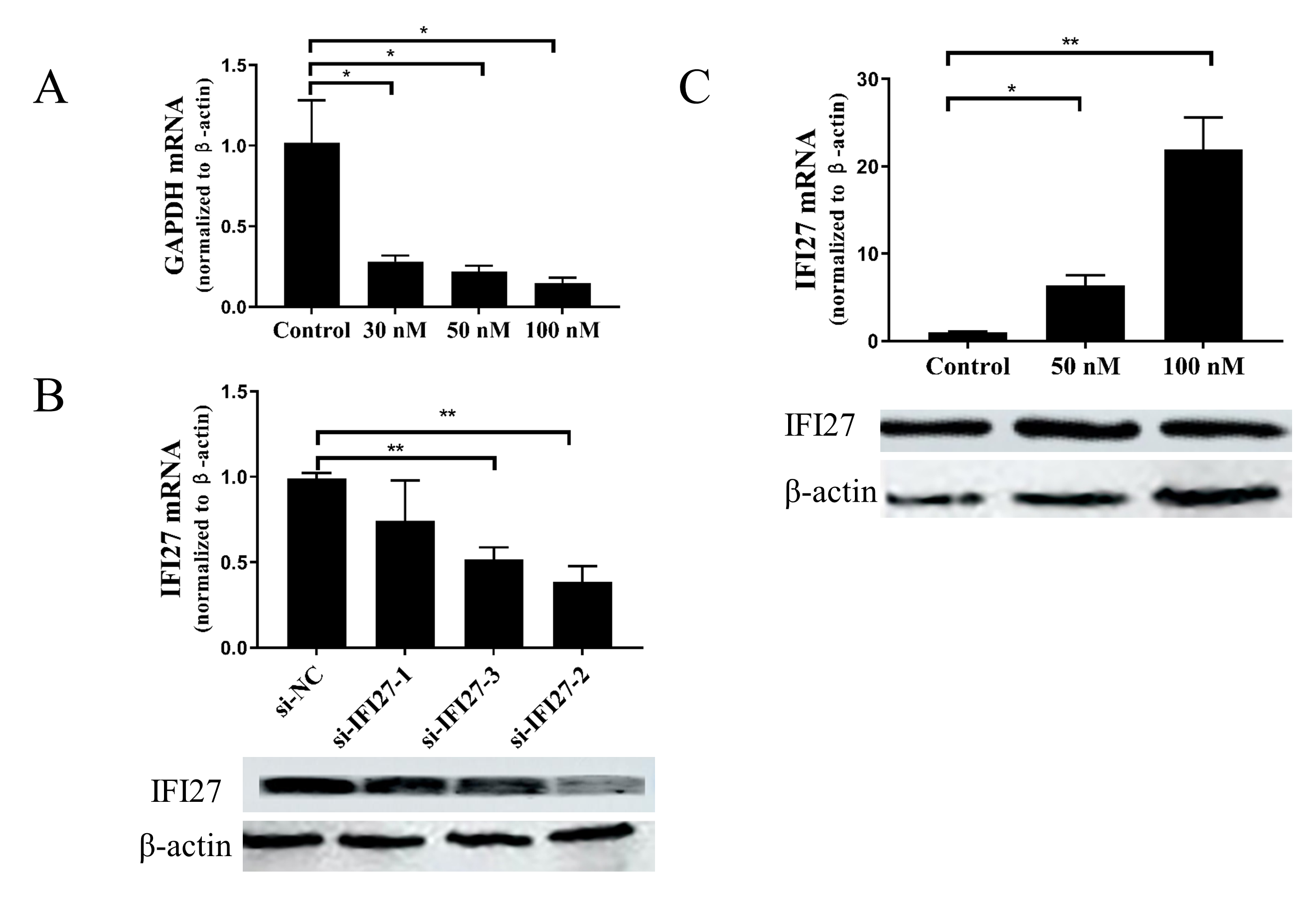

Supplement: Supplementary file 2 — Additional file 2: Figure S1. (A) Screening of siRNA transfection concentration, the transfectant was positive GAPDH siRNA. The optimal transfection concentration was 100 nM. (B) Screening of siRNA. si-IFI27-2 worked best. (C) Screening of plasmid transfection concentration. The optimal transfection concentration was 100 nM. All data represent the combined mean ± SD of three independent experiments with three technical replicates per experiment and were analyzed with a t-test vs. the controls. *P < 0.05; **P ≤ 0.01. [file 13071_2022_5415_MOESM2_ESM.tif]

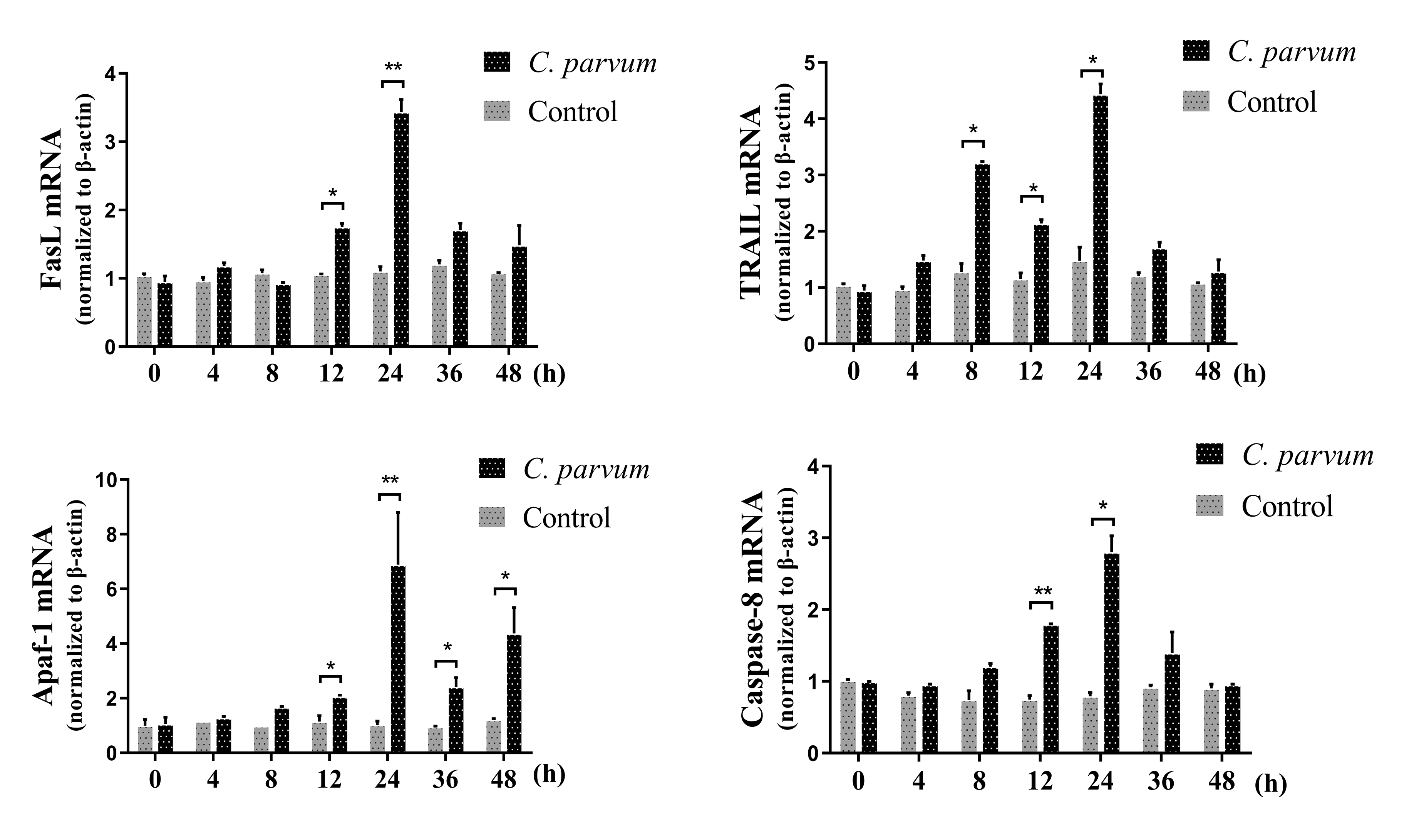

Supplement: Supplementary file 3 — Additional file 3: Figure S2. The level of apoptosis-related molecule expression at different time points after C. parvum infection. Cells were exposed to an equal number of C. parvum sporozoites for up to 48 h, followed by real-time PCR analysis for FasL, TRAIL, Apaf-1, and caspase-8 mRNA. All data represent the combined mean ± SD of three independent experiments with two to three technical replicates per experiment. The data were analyzed with a one-way ANOVA followed by a Dunnett’s test for multiple comparisons. *P < 0.05; **P ≤ 0.01; ***P ≤ 0.001; ****P ≤ 0.0001. [file 13071_2022_5415_MOESM3_ESM.tif]

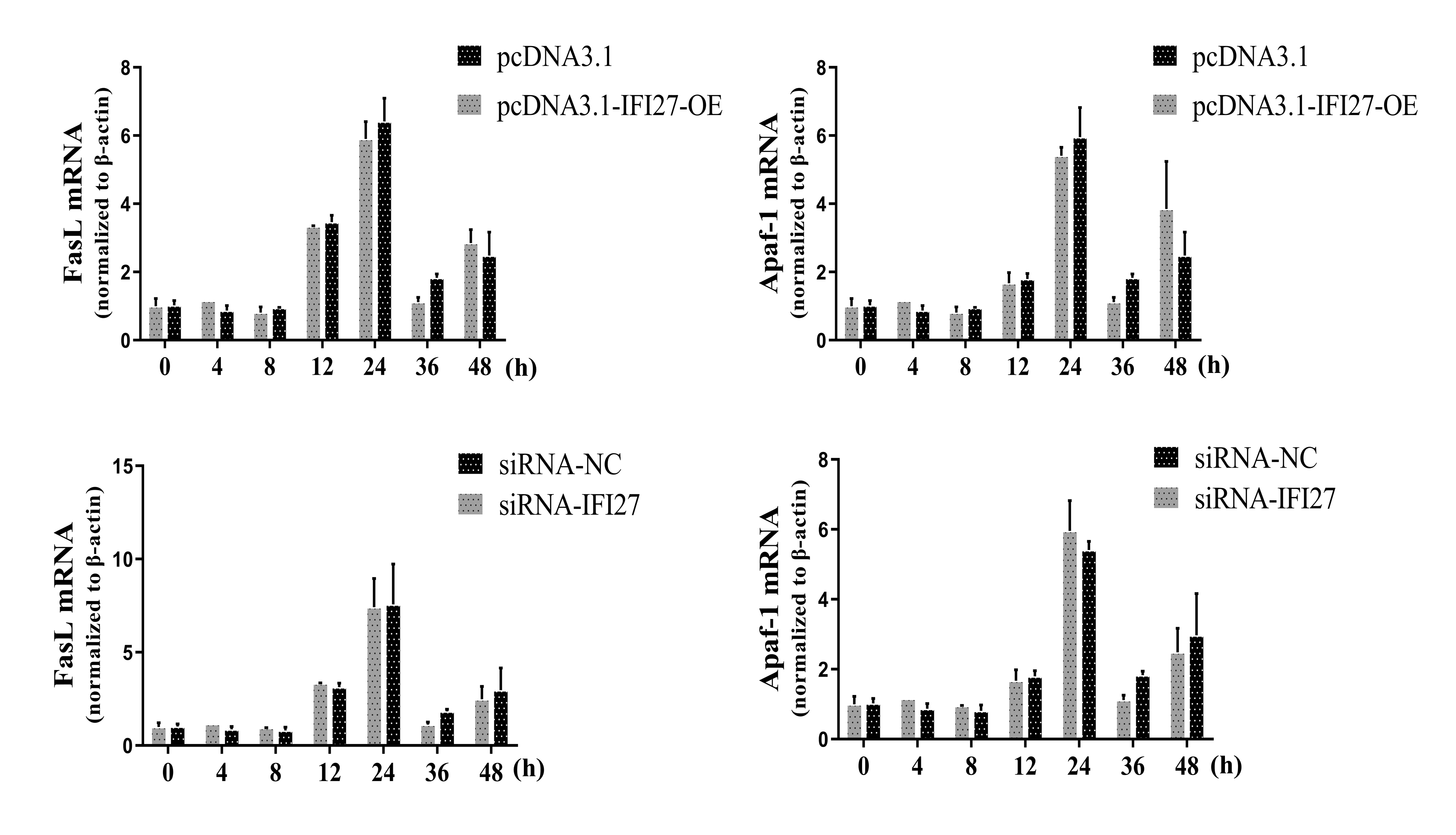

Supplement: Supplementary file 4 — Additional file 4: Figure S3. IFI27 does not regulate FasL and Apaf-1 expression after C. parvum infection in HCT-8 cells. Cells were transfected with pcDNA3.1-IFI27-OE or siRNA-IFI27 for 24 h. Cells were exposed to an equal number of C. parvum sporozoites for up to 48 h, followed by real-time PCR analysis for FasL and Apaf-1 mRNA. All data represent the combined mean ± SD of three independent experiments with two to three technical replicates per experiment. The data were analyzed with a one-way ANOVA followed by a Dunnett’s test for multiple comparisons. [file 13071_2022_5415_MOESM4_ESM.tif]
